# Quality of life for people with psychiatric disabilities employed in extended employment programs in two Arab towns in Israel: an exploratory study

**DOI:** 10.3389/fpsyt.2023.1307726

**Published:** 2023-12-22

**Authors:** Leena Badran, Stephen Rosenbaum, Arik Rimmerman

**Affiliations:** ^1^Department of Psychiatry and Behavioral Sciences, University of California, Davis, Sacramento, CA, United States; ^2^School of Law, University of California, Berkeley, Berkeley, CA, United States; ^3^School of Social Work, University of Haifa, Haifa, Israel

**Keywords:** Arab minority, community service, extended employment programs, psychiatric disabilities, quality of life, social norms

## Abstract

**Introduction:**

This study aims to examine the quality of life (QOL) for people with psychiatric disabilities who are engaged in extended employment programs (homogeneous versus heterogeneous) in the Arab-populated Triangle Area of Israel. The homogeneous program participants are exclusively Arab while the heterogeneous program includes both Arabs and Jews.

**Methods:**

Quantitative research study of 104 adults with psychiatric disabilities engaged in two communal extended employment programs. Participants completed demographic (age (years), gender, marital status (married, widowed/separated, married, single), religion (Muslim, Jewish, Christian), dichotomous nationality variable (Jewish/Arab), and years of education) and employment questionnaires (length of time in the employment program, number of working days/h and salary satisfaction); SF 12 Scale; and The Personal Wellbeing Index questionnaire. Two-sample *T*-Test, exploratory factor analysis and multiple linear regressions were conducted for tracking the differences between participants in homogeneous and heterogeneous programs.

**Results:**

A significant difference was found between the programs in two QOL components, insofar as satisfaction with the standard of living, together with health satisfaction were rated higher for participants in the heterogeneous program than for their homogeneous program counterparts. Furthermore, the results indicate that physical health and gender were the most important variables in explaining QOL in both programs, while the employment variables were not significant.

**Discussion:**

Since the research findings show that the employment-related-items aren’t significant in predicting the employees’ QOL, the definition and suitability of extended employment environments as a mental health service must be reexamined. Cultural elements may also have an impact on QOL when the programs are located in a traditional town, with gender playing a key role. The family’s role is pivotal in traditional societies, influencing an individual’s ability to participate in employment programs and the support they receive. In patriarchal societies, there can be added pressure on men with psychiatric disabilities to conform to societal expectations. Given the general lack of health awareness in Arab communities, there is a need to develop additional projects or incorporate physical health improvement as a rehabilitation goal when working with individuals with psychiatric disabilities, regardless of the type of community rehabilitation program.

## 1 Introduction

Employment rehabilitation for people with disabilities was initiated in Israel in the 1950s, beginning with sheltered workshops located alongside rehabilitation centers designed for acquisition of employment and social skills. These workshops were intended to simulate real workplace experiences. Community extended employment rehabilitation, not initiated until the 1980s, is a community-based, supportive employment setting for persons who wish to integrate into the labor market but presumably need to further develop their workplace skills. In most cases, this involves industrial work, with a range of 30–100 employees. Compensation is based on the quantity and quality of outputs. The staff consists of a social worker and rehabilitation instructors whose role is to assist participants in acquiring the necessary skills for advancement on the occupational ladder. Many of the individuals who participated in these controlled noncompetitive working environments consider them an important alternative to employment outside the hospital walls. These settings are nonetheless perceived as segregated and do not (necessarily) facilitate labor market integration (1).

The transition from a symptoms-focused to a community mental health approach mirrors the evolution from a medical model to a social or human rights model of disability. The social model explains disability, while the latter encompasses disability policy values and acknowledges the human dignity of persons with disabilities (2). In psychiatric rehabilitation, the medical model combines medical treatment with psychotherapy and hospitalization alternatives. In contrast, the (psycho)-social model emphasizes a holistic psychosocial view (3), focused on providing a wide range of community-based rehabilitation services, such as employment, housing, and leisure. These services, such as supported employment are intended to reduce the impact of disability and promote choice and empowerment (4).

This study examines the QOL among people with psychiatric disabilities employed in the first two extended employment programs (homogeneous versus heterogeneous) in the Triangle Area in Israel. The Triangle Area is a concentration of settlements in northern Israel, mainly populated by Muslim Arabs. About a fifth of the country’s Arab citizens live in this area. While one employment program employs only Arabs, the other employs both Arabs and Jews.

The World Health Organization (5) defines QOL as “individuals’ perception of their position in life in the context of the culture and value systems in which they live and in relation to their goals, expectations, standards and concerns” (p. 3). Use of the QOL concept, in the context of people with disabilities, began in the second half of the 20th century, when the biosocial paradigm arose as a result of the call for deinstitutionalization (6). It grew out of awareness of the environment’s effect on human behavior and the recognition that action must be taken to fulfill the lives of disabled persons.

Cummins and Lau (7) developed a model for measuring QOL by establishing a global group for personal well-being, whose main goal was to develop a global model for testing QOL that would be adapted to different cultures. Cummins (8) treated QOL as an objective, as well as subjective measurement. His model includes eight components: standard of living, health, life achievement, future security, safety, relationships, community connectedness and spirituality/religiosity.

The QOL among people with psychiatric disabilities is the subject of much research (9). Some believe that it should include the impact of the illness on individuals’ perception toward their QOL (10). Other studies have found that people with psychiatric disabilities report lower levels of QOL compared to people without psychiatric disabilities (4, 11). QOL related to health is actually a product indicator in light of the fact that, compared to the general population, people with psychiatric disabilities experience an increase in morbidity and early mortality (12). The QOL health index is seen as related to their psychiatric symptomatology (13, 14). Recently, researchers have distinguished between physical and mental health as related to QOL. Meijer et al. (14) reported in their research that the mental health related to QOL was stronger than that compared to physical health.

Employment is a significant element in individuals’ lives in income, social status and a person’s identity, social belonging, self-worth and physical and mental health (12). Previous research has shown that people with severe mental disorders and common mental disorders are, respectively, 7 and 3 times more likely to be unemployed than people with no disorders (14). More recent research has indicated the importance of meaningful employment for personal recovery from psychiatric disabilities (15).

People with psychiatric disabilities experience high rates of unemployment (15) and their workplace integration is a challenge. In addition to being economically disadvantaged, they are deprived of the social and psychological functions of work, such as social support, structured time, and self-esteem. Being without work and a stable income puts them at risk of poverty, even if they receive social security benefits (16). In addition, employment and economic satisfaction are associated with subjective positive QOL (17). Studies found a positive correlation between rehabilitation employment programs and QOL, among individuals with schizophrenia (18, 19). Although most people with psychiatric disabilities show a desire to work, they are more likely to experience adverse labor market outcomes than people without psychiatric disabilities (20).

Many programs have been developed to support people with psychiatric disabilities in their job search and integration into competitive employment. These are divided into (1) segregated skills training workshops and (2) supported employment in the general labor force (21). The first is the conventional and traditional model that aims to provide training and a transitional employment setting to prepare people for workforce integration. As an alternative option, supported employment is intended to place people in competitive employment with accompaniment. Empirical research demonstrates that supported employment has more positive consequences in the context of the number of working hours and salary than the traditional workshops (22, 23).

The employment rate among mentally ill people in Israel is lower compared to other OECD countries (14). Following global deinstitutionalization, a community rehabilitation law was enacted. The purpose of this law – the Rehabilitation in the Community of Persons with Mental Disabilities Law (2000) – is to allow people with psychiatric disabilities to be integrated into the community and promote functional independence and QOL, while maintaining their dignity in the spirit of the fundamental law of human dignity and freedom. The law defines a package of services to which persons with disabilities over age 18 are entitled, if they have a psychiatric disability at a rate of at least 40%, as determined by the National Insurance Institute, and “show motivation” to start the rehabilitation and recovery process in the community. The extended employment programs are part of the package of these community services.

Individuals who meet these criteria are allocated to one or more of these services through a committee comprised of psychiatric professionals, nurses, social workers, psychologists, and a representative from the rehabilitation staff designated by the Ministry of Health. Additionally, the individual is permitted to have family members participate in the committee discussions. Typically, this process concludes with a decision that is mutually agreed upon by all committee members, including the individual in question. An illustration of a decision made by this committee is the development of a comprehensive community rehabilitation plan for an individual. This plan encompasses three key components: an extended employment program, participation in a social club, and access to supportive education. This decision reflects the collaborative efforts of the committee members, who consider the individual’s specific needs and circumstances to create a tailored plan for their rehabilitation and overall well-being.

Comprising approximately 21% of the population, Arabs constitute the largest minority living in Israel. Living in a predominantly Hebrew-speaking, self-defined Jewish state, most Arabs are Muslim (85.1%) with the remainder being Christian (7.3%) and Druze (7.6%) (24). Arab society in Israel, as elsewhere, is characterized as traditional and patriarchal, emphasizing values such as family cohesion and social authority as a function of age, gender, and extended family status. The family provides the most obvious form of “natural support” (25) and is perceived as a significant source of financial and emotional sustenance. Within the family there is a hierarchy of gender and age; the man is the main breadwinner, and the woman is a secondary contributor to the household economy. The wife is expected to obey her husband and mainly take care of the children, and the latter are subordinate to both (26). Psychiatric disabilities may be considered as shameful and interpreted through a religious or supernatural lens (27). As a result, Arab families may tend to hide the mentally ill individual and avoid using the communal services for fear of revealing the disability. The status of Arabs with psychiatric disabilities is worsened by their belonging to a minority group that faces barriers such as insufficient health, unemployment, and poverty. These barriers in turn impair or heighten impairments to physical and mental health. On average, there is a higher prevalence of chronic diseases, higher incidence of disability and higher mortality rates among Arabs relative to the Jewish majority (28). Although the proportion of Arabs with disabilities is relatively higher than that of Jews, the availability of social services is lower.

An overview of psychiatric services in the Arab society in Israel reflects non-exhaustion of rights and underutilization of the few existing community-based services. Utilization of these services (within or outside Arab towns) is also low. This is a result of social stigma and the lack of cultural adaptation services in Arab communities. Underutilization of services stems in part from the cultural gap, but also from structural and systemic difficulties. Arab communities are mostly located in Israel’s geosocial periphery, and are characterized by limited access to public transportation, poor housing conditions, and lack of employment opportunities. Accordingly, the socioeconomic status of the Arab population is significantly disadvantaged relative to the Jewish population. Using services located outside of cities and towns involves effort, energy, and time. This includes difficulties in organizing transportation and overcoming the language barriers.

Individuals with mental illness (MI) are considered the most vulnerable group of disabled persons, having to cope with prejudice and severe social stigma (27). Huskin et al. (29), for example, demonstrated that persons with MI are perceived as inferior compared to those with physical disabilities. Regarding willingness to engage in relationships, they found that 30% of the participants would avoid being neighbors or socializing with co-workers with mental illness. Similar results were found in a study by Brockington et al. (30) that people with MI suffer the most from social rejection when compared to people with other disabilities. In most Arab countries, stigma towards mental illness is still widespread and people with MI continue to suffer from the impact of poverty and the stigma (31).

Furthermore, it’s important to acknowledge that in Arab communities, there can be a tendency to attribute psychiatric disabilities to factors such as a lack of faith and the influence of supernatural powers. This belief system often results in individuals failing to seek professional mental health assistance and underutilizing available mental health services, even when there is a clear need for such support. Despite the presence of a wide range of mental health services within Muslim society in Israel, we still observe an underutilization of these services.

For example, a survey conducted by the Myers-Joint-Brookdale Institute in Israel revealed that among Arabic speakers, a smaller proportion of individuals sought help from professional mental health services compared to informal sources of help. Specifically, the percentage of Arabic speakers aged 22 who reported seeking help from formal services was very low at 16%, especially when compared to Hebrew speakers (54%) and Russian speakers (34%). Additionally, a greater proportion of Arabic speakers (49%) sought assistance from informal agencies compared to Russian speakers (41%) and Hebrew speakers (26%) (32).

It is also essential to note that mental health research in these cultural contexts, particularly among Muslims in Israel, lags significantly behind, and there is a shortage of researchers focused on the field of mental illness within these communities. This gap is even more pronounced among minority groups with specific cultural beliefs about mental illness. Among Muslims, it is common to attribute mental illness to supernatural entities such as the demon Jinn, the evil eye Hasad, or magic Seher (33, 34). Additionally, there is a belief that mental illness can be caused by Allah, either as a punishment for sins or as a test, leading individuals to tolerate the condition and subject themselves to Allah’s will, potentially inhibiting their use of available treatments and community services. Psychiatric disabilities are often kept as a closely guarded secret within families. This is due to concerns about the potential negative repercussions, particularly in relation to the marriage prospects of other family members. This pervasive stigma can lead individuals with psychiatric disabilities to seek treatment from very expensive private psychiatrists.

The employment rehabilitation programs evaluated in the current study constitute the first controlled work environments for people with psychiatric disabilities in the Triangle Region. Both are located in different Arab communities but provide regional service and both programs were operated by an Israeli NGO. This organization aims to promote the progress and occupational and social integration of individuals with psychiatric disabilities within both the workplace and the community. This is achieved through rehabilitation offered in various occupational and social training centers. The approach encourages individuals to take an active role in their rehabilitation journey, conscientiously participating in the process, and assuming leadership in defining and pursuing their personal goals.

The current study has three hypotheses:

*Hypothesis 1:* Individuals with psychiatric disabilities participating in the heterogenous employment program have a higher level of QOL components than their counterparts in the homogeneous employment program.

*Hypothesis 2:* Physical health and gender have the most important impact on the eight QOL components of participants in the heterogenous employment program, while the employment conditions have no impact.

*Hypothesis 3:* Physical health, family status and gender have a significant impact on the eight QOL components, while employment conditions have impact mostly on the future security of participants in the homogeneous program.

## 2 Materials and methods

### 2.1 Participants and procedure

The quantitative research was approved by [Haifa University’s Faculty of Welfare and Health Sciences Ethics Committee]. It was conducted among Arabs and Jews with psychiatric disabilities employed in the first two extended employment programs in the Triangle Area in Israel.

Following approval from the Ministry of Health and from the NGO operating these two programs, the first author contacted the principal of each program asking for collaboration and for permission to attend the employees’ weekly meetings. These two employment programs are unique in the Triangle region as they are the sole providers of community occupational services for individuals with psychiatric disabilities. As stated above, all the employees in these programs meet the eligibility criteria outlined in the Rehabilitation in the (35). Specifically, individuals who are over 18 years old and have a psychiatric disability, as determined by the National Insurance Institute at a rate of at least 40%, are entitled to these services. Typically, these individuals spend the majority of their time in homes or institutions and possess limited employment skills. Given these criteria, all the employees in these programs were eligible to participate in the study.

Employees who agreed to participate in the study signed a preliminary informed consent form (with their guardian’s approval when required). The first author initiated contact with the guardians and provided them with an explanation of the study’s objectives. Subsequently, the guardians were invited to convene at the employment program location for a more comprehensive discussion and the formal signing of the consent form. These meetings were conducted with the participation of the guardian, the employee, and the first author to ensure clarity and understanding of the study’s details and to obtain their informed consent (10 guardians signed consent forms). Participants completed the questionnaire in their employment environment in a separate office. Those who encountered difficulties during the interview received assistance by having the question presented in simpler language.

Out of 135 employees in both programs, 104 agreed to participate in the study. Of the remaining 31 employees, 3 had declined, 23 were admitted for psychiatric hospitalization, and 5 had difficulty with completing the questionnaire due to their mental health condition. The heterogeneous program included 90 Arab and Jewish employees; the homogeneous program was composed of 45 Arabs.

Most of the participants were men (60%). More than half of them were single and 90% had a schizophrenia diagnosis. Most of them reported that they work 5 days a week. The average age of employees in the heterogeneous program is 40 ± 11 years, whereas in the homogeneous factory, it is 44 ± 11 years. In terms of marital status, the majority of those employed in the homogeneous factory are single, accounting for 58% of the workforce, as opposed to 64.3% in the heterogeneous factory. Additionally, none of the participants in either factory have attained a higher education level.

It’s also worth noting that all employees in the homogeneous factory reside in their own housing units or with their families within their local communities, which typically have a traditional housing structure. In contrast, within the heterogeneous enterprise, 70% of the participants come from housing frameworks outside of their family homes, while 24% live in more traditional housing arrangements, either with their nuclear families or with their parents (see Table 1).

**Table 1:** Sociodemographic characters of both employment program participants (*N* = 104).

Variables	Heterogeneous (*N* = 70)	Homogeneous (*N* = 34)	All participants (*N* = 104)
Gender			
Men	40 (57.1%)	23 (67.6%)	63 (60%)
Women	30 (42.9%)	11 (32.4%)	41 (40%)
Age (years)			
Range (R)	20–73	22–63	20–73
Mean	44.64	40	43.16
SD	11.52	11.18	11.5
Marital status			
Married	13 (18%)	8 (23.5%)	21 (20.1%)
Widowed	2 (2.8%)	1 (2.9%)	3 (2%)
Separated/divorced	10 (14%)	5 (14.7%)	15 (14.4%)
Single	45 (64.3%)	20 (58.8%)	65 (62.5%)
Education			
Range years (R)	1–19	3–20	1–20
Mean	9.28	10.5	9.67
SD	3.47	3.4	3.48
Religion			
Muslim	28 (40%)	34 (100%)	42 (59.6%)
Jewish	40 (57.1%)	–	40 (38.4%)
Christian	2 (2.9%)	–	2 (1.9%)
Housing type			
Live in my own apartment	11 (15.7%)	15 (44.1%)	36 (25%)
Lives in a rented apartment	1 (1.4%)	–	1 (0.09%)
Live with my parents	6 (8.6%)	19 (55.9%)	25 (24%)
Live in a hostel	41 (58.6%)	–	41 (39%)
Live in an assisted apartment	8 (11.4%)	–	8 (7%)
Hospitalized	3 (4.3%)	–	3 (2%)
Psychiatric diagnosis			
Schizophrenia	62 (88.6%)	28 (82.4%)	90 (86.5%)
Other	8 (11.45%)	6 (17.6%)	14 (13.4%)
Employment conditions			
Work experience			
Range (R)	10 (months)-4 (years)	10 (months)-4 (years)	10 (months)-4 (years)
Mean	2.18 years	1.7 years	2 years
SD	1.13	1.15	1.15
Working 5 days a week			
Yes	69 (98.6%)	34 (100%)	103 (99%)
No	1 (1.4%)	–	(0.9%)
Employment hours			
Range (R)	4–5	4–5	4–5
Mean	2.18 years	1.7 year	
SD	1.13	1.15	
Satisfaction of salary			
Range (R)	1–5	1–5	
Mean	4	4	
SD	1.4	1.49	

### 2.2 Instruments

To examine the independent variables, three questionnaires were used:

1. **The demographic questionnaire:** It included questions about the participants’ age (years), gender, marital status (married, widowed/separated, married, single), religion (Muslim, Jewish, Christian), dichotomous nationality variable (Jewish/Arab), and years of education.
2. **The employment conditions questionnaire:** This questionnaire included questions about the participant’s length of time in the employment program, whether they worked five days a week, their working hours and salary satisfaction.
3. **The health and mental health-related questionnaire** (short version of SF 12 Scale): It includes 12 items that measure functional status and is a shortened version of a 36-question questionnaire (SF-36). The original tool includes eight health concepts based on 36 items grouped into measures of physical health and mental health. The abbreviated tool includes 12 questions and is also grouped into two measures, physical and mental (36). The questionnaire examined satisfaction in eight health-related topics: physical functioning (PF) (Does your health now limit you in these activities such as climbing several flights of stairs?), role limitations due to physical health problems (RP) (During the past four weeks, have you had any of the following problems with your work or other regular daily activities as a result of your physical health?), bodily pain (BP) (How much did pain interfere with your normal work (including work outside the home and housework)?), general health (GH) (for example, How would you rate your health compared to your peers?), vitality (VT) (Did you have a lot of energy?), social functioning (SF) (During the past four weeks, how much of the time has your physical health or emotional problems interfered with your social activities (like visiting friends, relatives, etc.)?), role limitations due to emotional problems (RE) (During the past four weeks, have you had any of the following problems with your work or other regular daily activities as a result of any emotional problems (“feeling depressed” or “anxious”)?) and mental health (MH) (How much of the time during the past four weeks have you felt calm & peaceful?). Reliability in various studies was found between 0.7 and 0.89. The questionnaire was cross-translated for Arabic, Russian and Hebrew. Translation of the original SF12 questionnaire from English to Arabic was done by a translator in the King Fahd University Hospital Medical Education Center in Saudi Arabia, after receiving approval from the author of the questionnaire. In order to check the validity of the content of the translated version, three consultants from the hospital’s Department of Family and Community Medicine translated the questionnaire back into English, which was then cross-referenced with the original instrument. After a pilot study at a Saudi primary care center, with the participation of 20 diabetic patients, some wording was changed and words were added to improve the understanding of the questions. The test–retest reliability of the translated questionnaire was tested, and a Cronbach’s alpha of 0.84 was obtained. The test–retest reliability of the Arabic-translated questionnaire (37) was 0.84 Cronbach’s alpha. The questionnaire passed extensive reliability and validity tests. The reliability level of the subscales is high and ranges between 0.86–0.89 Cronbach’s alpha. In the current study an exploratory factor analysis, using the Varimax rotation method, was conducted and two factors were found: “physical health” and “mental health.” The reliability of the physical and mental aspects was 0.63,0.67, respectively, in this study.
4. **The Personal wellbeing index (PWI) questionnaire – adult** (38): This questionnaire was used to examine the dependent variable. **The PWI scale of Cummins** (7) contains eight items of satisfaction, each one corresponding to a QOL domain. One general question asks about standard of living (for example, How satisfied are you with your standard of living?), and seven additional individual questions ask about health (How satisfied are you with your health?), life achievement (How satisfied are you with what you are achieving in life?), relationships (How satisfied are you with your personal relationships?) safety (How satisfied are you with how safe you feel?), community-connectedness (How satisfied are you with feeling part of your community?), future security (How satisfied are you with your future security?), and spirituality/religion (How satisfied are you with your spirituality or religion?). These eight domains are theoretically embedded, as representing the first level deconstruction of the global question: How satisfied are you with your life as a whole?. Each question has an answer range from 1 to 7 (a score of 1 represents the lowest level). Cronbach alpha lies between 0.70 in Australia and 0.85 overseas. The translation of the questionnaire into Arabic was done in Algeria jointly with six senior lecturers in the field of social sciences at the University of Oran. The reliability of the questionnaire was more than 0.3 and Cronbach’s alpha more than 0.7 (39). In the current study, Cronbach’s alpha was 0.81.

### 2.3 Data analysis

Analyses were conducted with SPSS 27.0. Descriptive statistics were used to present the characteristics of the sample. T-Test for independent samples and exploratory factor analysis were used for assessing group differences. Different multiple linear regression models were run for each employment program. A *T*-Test for independent samples was employed to examine the statistical differences between the means of the two distinct groups. These groups exhibit notable differences from each other, and the dependent variable under investigation is the various components of QOL. This statistical test was chosen to assess whether there are significant differences in QOL components between the two groups.

In our study, exploratory factor analysis was performed using the principal component analysis with Varimax rotation. We hypothesized that a two-factor solution would be obtained with Eigen values greater than 1.

The multiple linear regression was carried out in two phases. In the first stage, correlation coefficient analyses (Spearman and Pearson) were conducted to examine the relationships between the independent variables and the QOL indicators. In the second stage, multiple linear regression analyses were performed for the variables that showed significant correlations.

## 3 Results

**Figure 1:**
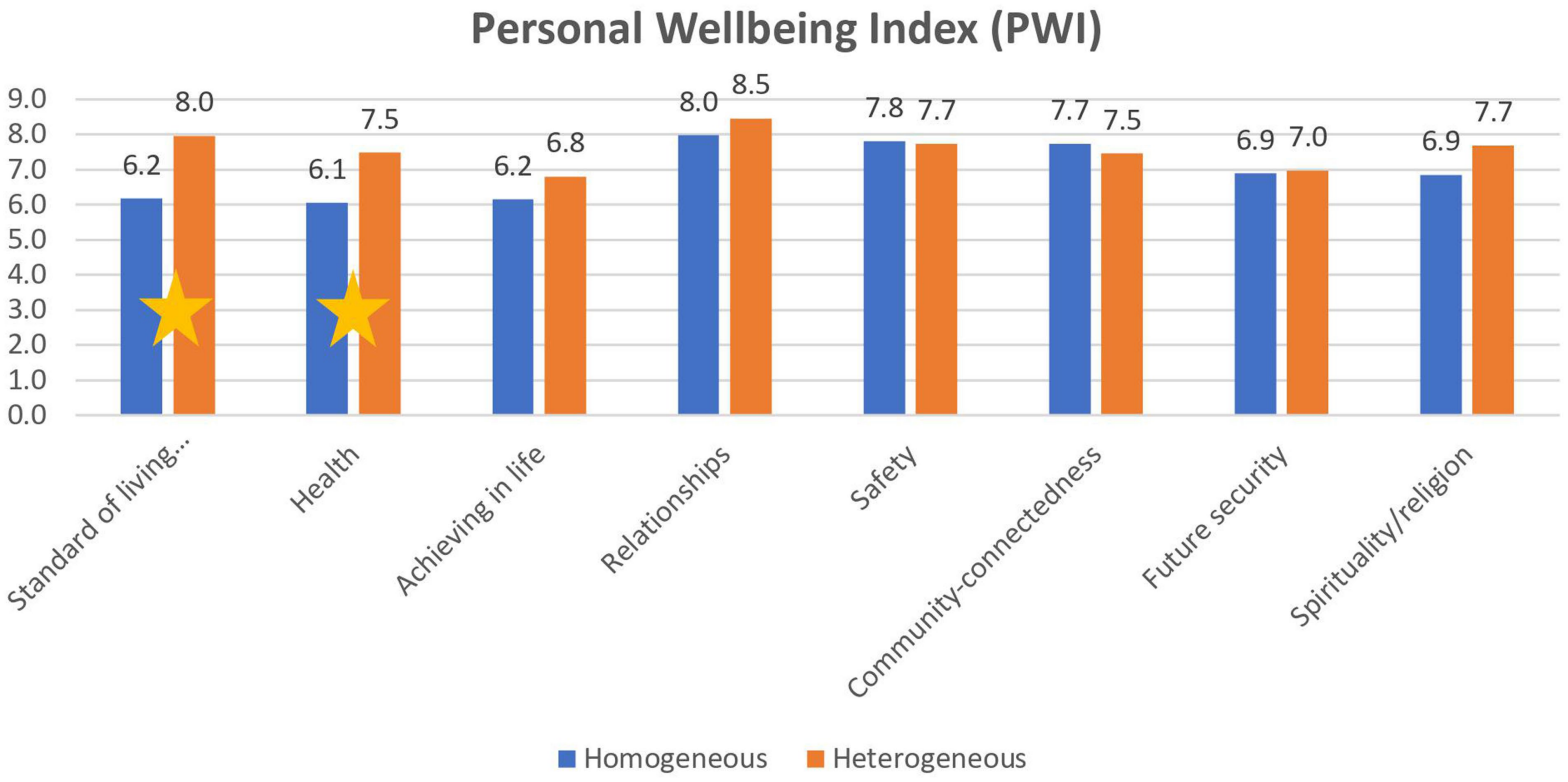
Differences in Personal Wellbeing Index (PWI) between the heterogeneous and the homogeneous programs.

*Hypothesis 1:* Individuals with psychiatric disabilities participating in the heterogenous employment program have a higher level of QOL components than their counterparts in the homogeneous employment program. *T*-Test for independence samples was conducted to examine this hypothesis and a significant difference was found in two components of QOL. A significant difference was found in satisfaction with the standard of living (t_(51.74)_ = −2.65, df = 51.74, *p* < 0.05). This was higher for those in the heterogeneous program than for those of the homogeneous program (*M* = 8, SD = 2.5, *M* = 6.2, SD = 3.5 respectively). A significant difference was also found in health satisfaction. The degree of health satisfaction among employees in the heterogeneous program (*M* = 7.5, SD = 3) was higher than for their homogeneous program counterparts (*M* = 6.1, SD = 3.8) (see Figure 1).

In order to examine whether subgroups of SF12 exist based on 12 items, an exploratory factor analysis was conducted using the principal component analysis (PCA) method for a sample of 104 participants, according to Eigen value of E.V < 1. An orthogonal Varimax rotation was also conducted. The results showed that physical functioning (PF), role limitations due to physical health problems (RP), bodily pain (BP) and general health (GH) items loaded higher on the physical component and vitality (VT), social functioning (SF), role limitations due to emotional problems (RE) and mental health (MH) loaded higher on the mental component. After the rotation, two factor structure (physical and mental) jointly accounted for 62.8% of the variance (See Table 2).

**Table 2:** Factor structure of the SF-12 derived from principal component analysis.

Rotated component matrix^*^		
	Component	
	1 Physical	2 Mental
General health (GH)		
General health evaluation compared to peers	**0.576**	−0.34
Physical functioning (PF)		
Limitations in moderate physical activities	**0.491**	0.18
Limitations in climbing several flights of stairs	**0.359**	0.15
Role physical (RP)		
Accomplished less due to physical health	**0.833**	0.066
Limited in kind of work or activities due to physical health	**0.540**	−0.076
Bodily pain (BP)		
Pain interferes with normal work	**−0.433**	−0.142
Role limitations due to emotional problems (RE)		
Accomplished less due to emotional problems	0.121	**0.887**
Not careful in work or activities due to emotional problems	0.162	**0.897**
Mental health (MH)		
Feel calm and peaceful	0.068	**−0.597**
Feel downhearted and blue	0.139	**0.791**
Vitality (VT)		
Having a lot of energy	−0.070	**−0.576**
Social functioning (SF)		
Interference of physical health or emotional problems with social activities	0.260	**0.589**

*Values equal or greater than 0.4 were considered satisfactory. Extraction method: principal component analysis. Rotation method: varimax with kaiser normalization. In this explanatory factor analysis, bold values indicate on which factor each item of SF 12 is loaded heavily. Specifically, the bold values below Factor 1 signify that the items with these bold values have higher loadings on Factor 1 compared to other factors. The results showed that GH,PF, RP and BP items loaded higher on the physical component and RE, MH, VT and SF loaded higher on the mental component.

*For hypotheses 2 and 3,* several linear regressions were conducted to test the impact of the sociodemographic variables, employment variables, and SF-12 components on the eight items of the QOL for each employment program. Pearson and Spearman tests were run to find the correlations between the dependent variable and the independent variables. Several regression models were run for variables with the significant correlations.

*Hypothesis 2:* It was hypothesized that Physical health and gender have the most important impact on the eight QOL components of participants in the heterogenous employment program, while the employment conditions have no impact. The linear regression showed that:

Mental component and employment hours explained 32% of the variance in health satisfaction [*F* = (5,56) = 6.8, *p* < 0.001]. The mental components variable is the most important in predicting health satisfaction (*β* = 0.81, *p* < 0.001). The positive coefficient indicates that the higher the level of mental component, the higher the degree of health satisfaction.

Gender explained 13% of the variance in community connectedness [*F* = (5,56) = 2.79, *p* < 0.05]. The gender variable is the most important in predicting a sense of community connectedness (*β* = 0.45, *p* < 0.001). Men have less sense of community connectedness.

Physical components and gender explained 16% of the variance of sense of future security [*F* = (5,56) = 3.39, *p* < 0.01]. The gender variable is the most important in predicting a sense of future security (*β* = 0.36, *p* < 0.01). Women have a greater sense of security in the future (see Table 3).

**Table 3:** Regression of the demographic variables and SF-12 for the heterogeneous employment program.

				Unstandardized coefficients	Standardized coefficients		
	Adjusted R square	*F*	Variables	*B*	Std. error	*β*	*t*
Standard of living	2%	1.3	N.A				
Health	32%	6.87^***^	Mental – SF-12	6.18	1.22	0.81^***^	5.04
			Employment hours	1.7	2.3	0.27^*^	2.01
Achieving in life	53%	0.38	N.A				
Relationships	7%	2.01	N.A				

Safety	6%	1.9	N.A				

Community-connectedness	13%	2.79^*^	Gender	2.76	0.8	0.45^***^	3.5
Future security	16%	3.39^**^	Physical SF-12	3.55	1.42	0.3^***^	2.5
			Gender	2.2	0.78	0.36^**^	−2.83
Spirituality/religion	2%	1.33	N.A				

*p < 0.05, **p < 0.01, ***p < 0.001.

*Hypothesis 3:* We hypothesized that physical health, family status and gender have a significant impact on the eight QOL components, while employment conditions have impact mostly on the future security of participants in the homogeneous program. Multiple linear regressions were conducted. The regression model is significant in the following variables:

Satisfaction of standard of living [*F*(6,22) = 7.19, *p* < 0.001]. The physical components variable is of the greatest importance in predicting satisfaction of standard of living (*β* = 0.53, *p* < 0.01). The higher the level of physical social functioning, the higher the level of satisfaction of standard of living.

Physical components, gender, age and family status explained 65% of the variance in health satisfaction [*F* = (5,24) = 11.8, *p* < 0.001]. The physical components variable is the most important in predicting health satisfaction (*β* = 0.81, *p* < 0.001).

Satisfaction of achieving in life [*F* = (5,24) = 2.9, *p* < 0.05]. The gender variable is the most important in predicting life achievement (β = −0.43, p < 0.05). Men were more satisfied than women with their achievements.

Satisfaction with relationships [*F* = (5,24) = 3.17, *p* < 0.05]. The physical components are the most important in predicting satisfaction with relationships (*β* = 0.6, *p* < 0.01) (see Table 4).

**Table 4:** Regression of the demographic variables and SF-12 for the homogeneous employment program.

				Unstandardized coefficients		Standardized coefficients	
	Adjusted R square	*F*	Variables	*B*	Std. Error	*β*	*t*
Standard of living	51%	7.19^***^	Physical – SF-12	1.740	2.03	0.53^**^	3.3
			Gender	2.70	1.4	0.35^*^	1.92
Health	65%	11.8^***^	Physical – SF-12	11.25	1.91	0.81^***^	5.88
			Age	0.14	0.05	0.39^*^	2.58
			Family status	−4.43	1.43	−0.48^***^	−3.09
			Gender	4.180	1.32	0.49^**^	3.15
Achieving in life	25%	2.9^*^	Physical – SF-12	4.7	2.6	0.36^*^	1.82
			Gender	−3.350	1.79	−0.43^*^	1.86
			Age	0.13	0.07	0.4^**^	1.83
Relationships	27%	3.17^*^	Physical – SF-12	7.04	2.33	0.6^**^	3.01
Safety	16%	2.100	N.A				
Community-connectedness	10%	1.690	N.A				
Future security	34%	4.090	N.A				
Spirituality/religion	16%	0.200	N.A				

*p < 0.05, **p < 0.01, ***p < 0.001.

## 4 Discussion

The current study examined the QOL among individuals with psychiatric disabilities employed in two extended employment programs. Several important findings are derived from our study:

### 4.1 Physical health and QOL among people with psychiatric disabilities

In both employment programs, the physical health was found to be a predictor of many of QOL components: Standard of Living, Future Security, Health, Life Achievement, Relationships. A positive relationship was found between the physical/mental health and QOL. Employees who highly rated their physical health also reported high levels of QOL in terms of overall satisfaction with health, a sense of community connectedness, and future security.

For people with psychiatric disabilities, QOL is based on social and economic independence, the freedom to function optimally, social and family support, and the right to mental and physical care (40). A person’s disability perception controls how he manages all areas of his life. In other words, as much as the person sees disability as a dominant part of his life, it will overshadow the other areas. Since the mortality rate among people with psychiatric disabilities is higher compared to the general population and also to people with other types of disabilities (41), their health conditions have impact on QOL (42). Vankova and Mancheva (43) found that the most influential QOL variant is the “Environment” domain, followed by the “Physical health,” “Psychological health,” and “Social relationships” domains, while the employment was not found to be a factor that affects QOL among people with disabilities.

### 4.2 Cultural aspects, gender issues, and psychiatric disabilities

The findings regarding gender presented interesting results. Women (in the heterogeneous program) were more satisfied, while men (in the homogeneous program) expressed a higher level of QOL. Significant differences between disabled women and men in their perceptions of QOL are documented in the literature (44). In the heterogenous programs, women reported higher levels of QOL than men, in terms of community connectedness and future security. Women in the homogeneous program reported a lower QOL in their standard of living, health, and life achievement. One possible explanation is that women employed in the extended employment program took on an additional role in their lives and graduated to the status of working women. Receiving a “salary” may increase their social status in traditional Arab society. This, in return, increases the sense of belonging to life in the community or neighborhood. In this role, women are exposed to various relationships, perceiving this environment as a place where they can engage in social networking. It is therefore likely that they will report a high level of social ties and belonging (45).

Women had diverse social roles in their personal lives due to program participation, which, in turn, increased their self-esteem and reduced preoccupation with their disability. Arab women are also more likely to live with families and acquaintances, leading to higher satisfaction in terms of social belonging. In addition, women employed in the heterogeneous program were exposed to a diverse social network. Arab women made connections with Jewish women, the latter belonging to the majority society which is considered “modern” and “developed.” These connections may very well raise the self-esteem and feelings of appreciation and future security.

In the homogeneous program, men reported higher levels of QOL than did women. In the homogenous program, located in a conservative town, the traditional patterns favoring men were preserved. That is, the attitude of others towards women with disabilities is protective and even domineering. This reduces possibilities for women and emphasizes their disability. The reduction of their freedom of choice, as well as the controlling and restrictive social attitude, seem to explain differences in the QOL scores of women and men in the current study. This finding raises a question for future research as to whether there is an effect of the integration of Arabs and Jews in the employment environment on their QOL?

Regarding marital status, unmarried employees in the homogenous program reported a higher sense of safety and standard of living than married employees. This stands in contrast to the study by Trompenaars et al. (45) which found participants involved in a partner relationship had significantly higher scores in all domains of the QOL. It can be argued that there may be an effect on social norms regarding gender roles, insofar as a married man is expected to bear the burden of his family’s financial support, which, in turn, may increase emotional stress. In addition, single employees have fewer social and economic commitments. It is possible that their public social insurance benefits, together with the rehabilitation compensation they receive for work in the employment program, allows them to live without feeling pressure and concern for others. Quality of the marriage may also affect the sense of safety and living standard (46).

### 4.3 Employment factors and QOL

The number of working hours is the only variable related to employment conditions that has an impact on health satisfaction in the heterogeneous context. Given limited opportunities in this region, the extended employment program was the first opportunity for most of the participants to reestablish good work habits. According to the literature, the working hours in an extended environment has an effect on the employee’s QOL (22). The findings reveal that employees with longer working hours (5 h) expressed a higher degree of satisfaction with health than their counterparts who worked fewer hours (4 h). Accordingly, employees who worked more hours per day perceived themselves as having a greater influence, given their longer hours of employment and greater productivity. Furthermore, employees who managed to work 5 h in the workplace were rewarded for their commitment progress. As a result, these employees will tend to see themselves as achievers and will have a high sense of personal security. In terms of spiritual and religious satisfaction, the findings did not indicate any impact on QOL.

### 4.4 Research and applied implications

This exploratory study has several important research implications. First, it presents preliminary findings on the population of Arabs with psychiatric employed in an extended employment program, and the effect of the employment environment—homogeneous vs. heterogeneous—on their QOL. Future research should examine the impact of Arabs and Jews working in the same community-based employment environment on their QOL.

Research should also include a longitudinal study that will follow reported changes over time, as opposed to this cross-sectional study. Since psychiatric disabilities has many fluctuations along the timeline, it is important to conduct several measurements. Lastly, the cultural dimension must continue to be considered when studying the Arab or Muslim social milieu, since families in traditional society have an important role in providing support to persons with psychiatric disabilities (25, 47), including their involvement in decision-making. It is also important to examine the family as a research variable. The extended employment programs is perceived by many families as a place through which they can be freed from the burden imposed on them, by the fact that the family member with a psychiatric disability is away from home for several hours. The quality of life of the employment staff should also be studied.

As to policymakers, there is a general lack of awareness in Arab society, including among people with psychiatric disabilities, regarding the importance of maintaining a healthy lifestyle. Additionally, behaviors such as excessive smoking, side effects associated with psychiatric medications, and insufficient support from family members contribute to this issue.

There is a pressing need for collaboration between policymakers and organizations involved in community rehabilitation services to develop programs and initiatives aimed at enhancing the physical health of individuals with psychiatric disabilities. Many rehabilitation programs already provide companions to accompany individuals to medical and psychiatric appointments. These companions should receive training to incorporate additional components related to physical health within the rehabilitation process. Improving physical health can have a profound impact on overall performance and quality of life.

### 4.5 Limitations

This study has several limitations. The research was only carried out in two extended employment programs in Arab towns in the center of the country, and its findings refer only to the people employed in these two workplace settings. Since this is an exploratory study of a relatively small population, it is difficult to generalize to the entire population of those mentally disabled individuals employed in extended employment programs in Israel.

## 5 Conclusion

Since the research findings show that the employment-related-items aren’t significant in predicting the employees’ QOL, the definition and suitability of extended employment environments as a mental health service must be reexamined. In both employment programs, the physical health was found to be a predictor of many of QOL components: Standard of Living, Future Security, Health, Life Achievement, Relationships. Our finding emphasizing that physical health needs to be studied and improved due to its impact on QOL for people with psychiatric disabilities in multiple areas.

## Data availability statement

The original contributions presented in the study are included in the article/supplementary material, further inquiries can be directed to the corresponding author.

## Ethics statement

The studies involving humans were approved by Faculty of Social Welfare and Health Sciences-Ethic committee-Haifa University. The studies were conducted in accordance with the local legislation and institutional requirements. Written informed consent for participation in this study was provided by the participants’ legal guardians/next of kin. Written informed consent was obtained from the individual(s) for the publication of any potentially identifiable images or data included in this article.

## Author contributions

LB: Conceptualization, Data curation, Formal analysis, Investigation, Methodology, Project administration, Software, Validation, Visualization, Writing – original draft, Writing – review & editing. SR: Conceptualization, Validation, Writing – review & editing. AR: Conceptualization, Investigation, Methodology, Supervision, Validation, Writing – review & editing.

## References

[1] Schwartz C Armony-Sivan R. Students’ attitudes to the inclusion of people with disabilities in the community. Disabil Soc. (2001) 16:403–13. doi: 10.1080/09687590120045978

[2] Rosenbaum SA. Restoring voice to people with cognitive disabilities: realizing the right to equal recognition before the law. J Leg Med. (2019) 39:61–74. doi: 10.1080/01947648.2019.1587653

[3] Janice C John B Alicia O Myfanwy L Suzy P. Quality of life of people with mental health problems: a synthesis of qualitative research. Heal Qual Life Outcomes. (2012) 10:1–16. doi: 10.1186/1477-7525-10-138 PMC3563466 23173689

[4] Berghöfer A Martin L Hense S Weinmann S Roll S. Quality of life in patients with severe mental illness: a cross-sectional survey in an integrated outpatient health care model. Qual Life Res. (2020) 29:2073–87. doi: 10.1007/s11136-020-02470-0, PMID: 32170584 PMC7363717

[5] Organization WH (1997) Measuring quality of life: The World Health Organization quality of life instruments. Geneva.

[6] Erez A Gal E. Quality of life: a universal or a disability specific concept? Can J Occup Ther. (2020) 87:4–11. Available from:. doi: 10.1177/0008417419831552 31795727

[7] Cummins RA Lau ALD. An introduction to the international wellbeing group and the international wellbeing index. Fifth conference of the International Society for Quality-of-Life Studies: Frankfurt, Germany (2005).

[8] Cummins R. Personal well-being index. 4th ed. Melbourne: Australian Centre on Quality of Life, Deakin University (2006).

[9] Barnes AL Murphy ME Fowler CA Rempfer MV. Health-related quality of life and overall life satisfaction in people with serious mental illness. Schizophr Res Treatment. (2012) 2012:1–6. doi: 10.1155/2012/245103, PMID: 23213525 PMC3507053

[10] Lee RSC Hermens DF Naismith SL Lagopoulos J Jones A Scott J. Neuropsychological and functional outcomes in recent-onset major depression, bipolar disorder and schizophrenia-spectrum disorders: a longitudinal cohort study. Transl Psychiatry. (2015) 5:e555–5. doi: 10.1038/tp.2015.50, PMID: 25918992 PMC4462613

[11] Folsom DP Depp C Palmer BW Mausbach BT Golshan S Fellows I. Physical and mental health-related quality of life among older people with schizophrenia. Schizophr Res. (2009) 108:207–13. doi: 10.1016/j.schres.2008.12.008, PMID: 19168328 PMC3643118

[12] Meijer CJ Koeter MWJ Sprangers MAG Schene AH. Predictors of general quality of life and the mediating role of health related quality of life in patients with schizophrenia. Soc Psychiatry Psychiatr Epidemiol. (2009) 44:361–8. doi: 10.1007/s00127-008-0448-4, PMID: 18974910

[13] Blustein DL. The role of work in psychological health and wellbeing – a conceptual, historical and public policy perspective. Am Psychol. (2008) 63:228–40. doi: 10.1037/0003-066X.63.4.228, PMID: 18473608

[14] OECD (2012) Sick on the job? Myths and realities about mental health and work. Paris, France: OECD Publishing.

[15] Kinoshita Y Furukawa TA Kinoshita K Honyashiki M Omori IM Marshall M. Supported employment for adults with severe mental illness. Cochrane Database Syst Rev. (2013) 2013:CD008297. doi: 10.1002/14651858.CD008297.pub2, PMID: 24030739 PMC7433300

[16] Rüesch P Graf J Meyer PC Rössler W Hell D. Occupation, social support and quality of life in persons with schizophrenic or affective disorders. Soc Psychiatry Psychiatr Epidemiol. (2004) 39:686–94. doi: 10.1007/s00127-004-0812-y 15672288

[17] Hoffmann H Kupper Z Zbinden M Hirsbrunner H-P. Predicting vocational functioning and outcome in schizophrenia outpatients attending a vocational rehabilitation programme. Soc Psychiatry Psychiatr Epidemiol. (2003) 38:76–82. doi: 10.1007/s00127-003-0603-x, PMID: 12563549

[18] Bryson G Lysaker P Bell M. Quality of life benefits of paid work activity in schizophrenia. Schizophr Bul. (2002) 28:249–57. doi: 10.1093/oxfordjournals.schbul.a006935, PMID: 12693431

[19] Hampson ME Hicks RE Watt BD. Perspectives on the benefits and costs of employment and unemployment for people living with psychosis. Am J Psychiatr Rehabil. (2018) 21:355–80.

[20] Carmona V Gómez-Benito J Huedo-Medina T Rojo J. Employment outcomes for people with schizophrenia spectrum disorder: a meta-analysis of randomized controlled trials. Int J Occup Med Environ Health. (2017) 30:345–66. doi: 10.13075/ijomeh.1896.01074 28481370

[21] Crowther RE. Helping people with severe mental illness to obtain work: systematic review. BMJ. (2001) 322:204–8. doi: 10.1136/bmj.322.7280.204, PMID: 11159616 PMC26585

[22] Bailey E Ricketts S Becker D Xie H Drake R. Do long-term day treatment clients benefit from supported employment? Psychiatr Rehabil J. Disability employment TA Center (1998) 22:24–9. doi: 10.1037/h0095270

[23] Schwartz S Ruby M (2021) Ending segregated workshops and promoting competitive, integrated employment (CIE). Disability employment TA Center.

[24] Badran L Amin H Gur A Stein M. ‘I am an Arab Palestinian living in Israel with a disability’: marginalisation and the limits of human rights. Disabil Soc. (2023):1–22. doi: 10.1080/09687599.2023.2181764

[25] Rosenbaum S. Legal capacity’ under the UN convention on the rights of persons with disabilities: In Support of Supported Decision-Making Islam Stud Hum Rights Democr (2023). 3 p.

[26] Gur A Gnaeem-Badran L Stein MA. The role of grandparents in Israeli Muslim families with intellectually disabled fathers: social workers’ perspectives. Soc Work. (2021) 66:139–47. doi: 10.1093/sw/swab006, PMID: 33855458

[27] Badran L Rimmerman R. Muslim social workers and imams’ recommendations in marital and child custody cases of persons with intellectual or mental disability. Br J Soc Work. (2021) 52:1249–68. doi: 10.1093/bjsw/bcab137/6327447

[28] Knesset. People with disabilities in Arab society: General data and data on students in the special education system. Research and Information Center: Knesset (2017).

[29] Huskin PR Reiser-Robbins C Kwon S. Attitudes of undergraduate students toward persons with disabilities: exploring effects of contact experience on social distance across ten disability types. Rehabil Couns Bull. (2018) 62:53–63. doi: 10.1177/0034355217727600

[30] Brockington I Hall P Levings J Murphy C. The Community’s tolerance of the mentally ill. Br J Psychiatry. (1993) 162:93–9. doi: 10.1192/bjp.162.1.93, PMID: 8425146

[31] Abi Doumit C Haddad C Sacre H Salameh P Akel M Obeid S. Knowledge, attitude and behaviors towards patients with mental illness: results from a national Lebanese study. PLoS One. (2019) 14:e0222172. doi: 10.1371/journal.pone.0222172, PMID: 31525219 PMC6746362

[32] Knesset (2014). Mental health clinics in the Arab communoty, research and information center. Available at: https://fs.knesset.gov.il/globaldocs/MMM/e7556b58-e9f7-e411-80c8-00155d010977/2_e7556b58-e9f7-e411-80c8-00155d010977_11_10016.pdf (Accessed November 9, 2022)

[33] Al-Adawi S Dorvlo A Al-Ismaily S Al-Ghafry D Al-Noobi B Al-Salmi A. Perception of and attitude toward mental illness in Oman. Int J Soc Psychiatry. (2002) 48:305–17. doi: 10.1177/002076402128783334, PMID: 12553410

[34] Al-Issa I. Al-Junun: Mental illness in the Islamic world. International Universities Press (2000).

[35] Community Rehabilitation of Persons with Mental Health Disability Law. (2000a). LB 1746 (Hebrew). Available at: https://www.health.gov.il/LegislationLibrary/Nefesh35.pdf

[36] Ware JE Kosinski M Keller SD. A 12-item short form health survey: construction of scales and preliminary tests of reliability and validity. Med Care. (1996) 34:220–33. doi: 10.1097/00005650-199603000-00003 8628042

[37] Al-Shehri A Taha A Bahnassy A Salah M. Health-related quality of life in type 2 diabetic patients. Ann Saudi Med. (2008) 28:352–60. doi: 10.5144/0256-4947.2008.352, PMID: 18779640 PMC6074492

[38] Cummins RA. Moving from the quality of life concept to a theory. J Intellect Disabil Res. (2005) 49:699–706. doi: 10.1111/j.1365-2788.2005.00738.x 16162114

[39] Tiliouine H Cummins R Davern M. Measuring wellbeing in developing countries: the case of Algeria. Soc Indic Res. (2006) 75:1–30. doi: 10.1007/s11205-004-2012-2

[40] Corrigan PW Watson AC. Understanding the impact of stigma on people with mental illness. World Psychiatry. (2002) 1:16–20. PMID: 16946807 PMC1489832

[41] Segal SP Badran L Rimes L. Accessing acute medical care to protect health: the utility of community treatment orders. Gen Psych. (2022) 35. Available from::e100858. doi: 10.1136/gpsych-2022-100858, PMID: 36654668 PMC9764604

[42] Fitzgerald PB Williams CL Corteling N Filia SL Brewer K Adams A. Subject and observer-rated quality of life in schizophrenia. Acta Psychiatr Scand. (2001) 103:387–92. doi: 10.1034/j.1600-0447.2001.00254.x, PMID: 11380309

[43] Vankova D Mancheva P. Quality of life of individuals with disabilities – concepts and concerns. Scr Sci Salut Publicae. (2015) 1:21–44. doi: 10.14748/sssp.v1i1.1151

[44] Dernovsek MZ. The quality of life of schizophrenia outpatients. Eur Psychiatry. (1997) 12:223s–32s. doi: 10.1016/S0924-9338(97)80704-3

[45] Trompenaars FJ Masthoff ED Van Heck GL Hodiamont PP De Vries J. Relationships between demographic variables and quality of life in a population of Dutch adult psychiatric outpatients. Soc Psychiatry Psychiatr Epidemiol. (2005) 40:588–94. doi: 10.1007/s00127-005-0946-0, PMID: 16021343

[46] Xiang Y-T Wang C-Y Wang Y Chiu HFK Zhao J-P Chen Q. Socio-demographic and clinical determinants of quality of life in Chinese patients with schizophrenia: a prospective study. Qual Life Res. (2010) 19:317–22. doi: 10.1007/s11136-010-9593-9, PMID: 20135234

[47] Badran L Hejazi A Rimmerman A. Muslim social workers’ recommendation of parents with mental illness or intellectual disability disorders in vignettes of simulated religious court cases of custody, marriage, and divorce. J Relig Spiritual Soc Work Soc Thought. (2022) 42:1–21. doi: 10.1080/15426432.2022.2160409

